# Viability, yield and expansion capability of feline MSCs obtained from subcutaneous and reproductive organ adipose depots

**DOI:** 10.1186/s12917-021-02948-0

**Published:** 2021-07-15

**Authors:** Amy Wysong, Priscilla Ortiz, Douglas Bittel, Lindsey Ott, Francis Karanu, Michael Filla, Lisa Stehno-Bittel

**Affiliations:** 1 College of Biology, Kansas City University, 1750 Independence Ave, Kansas City, MO USA; 2 Innova Celulas Madre, San Pedro, San Jose, Costa Rica; 3 Likarda LLC, 10330 Hickman Mills Drive, Kansas City, MO USA; 4 Department of Rehabilitation Science, University of Kansas Medical Center, 3901 Rainbow Blvd, KS 66160 Kansas City, USA

**Keywords:** Feline, MSC, Multipotent stem cells, Expansion, Gata6, Sox2, Sox17

## Abstract

**Background:**

The source of multipotent stromal cells (MSC) can have a significant influence on the health and expansion capacity of the cells. As the applications for allogeneic MSCs in the treatment of feline diseases increase, the location of the initial donor tissue must be analyzed. To date, comparisons have only been made between feline MSCs collected from bone marrow or abdominal fat. This is the first report to compare cells obtained from different adipose depots in the cat *with a focus on clinically relevant donor tissues*. The tissue was collected from *34* healthy cats undergoing spaying (fat around the ovaries and uterine horn) or subcutaneous fat collected during surgical procedures.

**Results:**

The amount of starting material is essential to isolate sufficient MSCs. The total tissue yield from the subcutaneous fat was significantly greater than could be obtained from around the reproductive organs, leading to 3 times more MSCs per donor. However, the concentration of MSCs obtained from reproductive fat was higher than from subcutaneous fat. In addition, the viability of the MSCs from the reproductive fat was significantly higher than the subcutaneous fat. Since most spaying occurs in young cats (under 18 months) reproductive fat was collected from adult cats during spaying, illustrating that age did not alter the yield or viability of the MSCs. When sufficient tissue was collected, it was digested either mechanically or enzymatically. Mechanical digestion further decreased the viability and yield of MSCs from subcutaneous fat compared to enzymatic digestion. Biomarkers of stem cell characterization, expansion capacity and function were detected using qPCR. CD70, CD90 and CD105 were all expressed in high levels in the 3 groups. However, the reproductive fat had higher levels of CD73 with the mechanically digested subcutaneous fat having the least. Gata6 was detected in all samples while Sox2 and Sox17 were also detected with higher quantities found in the enzymatically digested subcutaneous fat. Negative control genes of Gata4 and Pdx1 showed no detection prior to 50 cycles. During the first three passages, age of the donor, location of the donor tissue, or digestion protocol had no effect on cell culture doubling times or cell viability.

**Conclusions:**

While MSCs from reproductive fat had superior cells/tissue weight and initial viability, there were still dramatically fewer cells obtained compared to subcutaneous fat due to the limited amount of tissue surrounding the reproductive organs. Further, in P1-P3 cultures there were no differences noted in doubling time or cell viability between tissue obtained from reproductive or subcutaneous fat depots.

**Supplementary Information:**

The online version contains supplementary material available at 10.1186/s12917-021-02948-0.

## Background

Multipotent stromal cells (MSC) are increasingly popular in regenerative medicine because of the ease of access and isolation, ability to self-renew and multipotent potential [1]. These adult stem cells have the ability to differentiate into cells of the mesodermal lineage such as adipocytes and osteoblasts, but they also have transdifferentiation properties allowing them to be driven to other cell types such as neurons or myocardial cells [2]. Reports have described the therapeutic application of MSCs to treat various conditions such as heart failure, inflammatory diseases, musculoskeletal trauma, diabetes and kidney disease [3]. Additionally, MSCs have successfully been used for treating inflammatory conditions such as osteoarthritis or graft versus host disease [4].

There are numerous clinical applications for MSCs in the veterinary field, including the treatment of feline diseases such as gingivostomatitis, inflammatory bowel disease, allergic asthma and chronic kidney disease [5–8]. Research on MSC-based treatment for feline ailments is expanding our understanding of the potential of stem cells, along with the challenges associated with feline cells.

MSCs can be harvested from a variety of sources including adipose tissue, bone marrow, peripheral blood, and umbilical cord [9, 10]. Cells from diverse sites have unique properties including basic transcriptome differences and variations in *in vitro* or *in vivo* differentiation [11]. Of the multiple sites from which to obtain MSCs, adipose tissue is one of the most commonly used and widely studied. Historically, adipose tissue was considered a simple site of energy storage, but this is no longer the case. Adipose tissue is a functionally dynamic organ that is involved in energy homeostasis and the secretion of several hormones [12].

The abdominal cavity includes several types of adipose tissue, such as subcutaneous, intramuscular and visceral. Subcutaneous adipose tissue lies underneath the skin and is composed of adipocytes that are organized in lobules, separated by connective tissue. Subcutaneous adipose tissue makes up about 80 % of all body fat [13]. In contrast, visceral adipose tissue is interspersed among the organs and composed of several different depots including mesenteric, perirenal, and fat surrounding the reproductive organs. The MSC characteristics can vary depending on the site of tissue harvest [14]. Reports demonstrate differences in MSC characteristics when comparing subcutaneous and visceral adipose tissue surrounding female reproductive tissue obtained during Caesarean Sections. [15, 16]. Ritter et al. concluded that human visceral fat associated with the reproductive organs had a higher potential to differentiate into adipogenic and osteogenic cells and secreted higher levels of inflammatory cytokines, emphasizing the importance of the site of tissue harvest [15]. Likewise, research in felines has suggested that reproductive fat may have advantages over other fat depots [17].

While studies have compared feline MSCs obtained from either bone marrow or fat tissue [18], no study has compared the different abdominal depots of fat for MSC retrieval in cats. When considering the translation of this research to a scaled MSC-based product, we determined that the tissue source should be easily retrieved as part of standard veterinary practice from community-dwelling cats when possible. The objective of the study was to compare feline adipose tissue from subcutaneous and reproductive organ donor sites to test the hypothesis that enzymatically digested reproductive fat would result in higher levels of expansion and multipotent biomarkers along with higher viability and proliferation capabilities.

## Results

### Donor Characteristics

The study design was a non-randomized subject sample of convenience with tissue collection occurring secondary to standard veterinary clinic procedures. 94 % of the procedures were conducted at licensed veterinary clinics on their clients or at non-profit animal shelters prior to adoption. Only 2 cats were part of research colonies with fat retrieved during procedures related to the other research studies in which they were enrolled.

The average age of the donors in the reproductive group was younger than the subcutaneous group due to the fact that most spaying occurs in pediatric cats. However, 7 spayed cats in the reproductive fat group were over 18 months and thus results of the reproductive fat group were subdivided into pediatric and adult cats to determine whether the age of the donors affected the results. Since all veterinary procedures that allowed the collection of subcutaneous fat occurred on adult cats, there was no pediatric equivalent group. There was no difference in the mean age of the adult cats in the adult reproductive and subcutaneous tissue groups (Table 1). The average body weight was less for the pediatric reproductive group compared to the two adult donor groups (Table 1). Likewise, the body condition scores were statistically greater in the adult subcutaneous tissue donors compared to the other groups (Table 1).

**Table 1:** Summary of donor characteristics. Fat samples were obtained from healthy cats with the following characteristics.

	Pediatric Reproductive Tissue	Adult Reproductive Tissue	Adult Subcutaneous Tissue
Age (years)	0.38 ± 0.06 #	3.68 ± 0.85	3.81 ± 1.90
Sex (% female)	100 %	100 %	29 %
Donor Body Weight (kg)	2.20 ± 0.02 #	3.57 ± 0.32	4.50 ± 0.44
Body Condition Scores (1 to 5)	3.44 ± 0.31	3.43 ± 0.20	4.50 ± 0.20 *
Average Tissue Weight (gm)	0.40 ± 0.06	0.34 ± 0.29	9.00 ± 1.84 *
Range of Tissue Weight (gm)	0.22–0.59	0.08–0.53	4.21–12.44 *
Total Cells (in million)	2.22 ± 0.69	2.82 ± 1.43	6.03 ± 0.54 *

* statistically significant differences between adult subcutaneous tissue group and reproductive tissue groups (*p* < 0.05)

# statistically significant differences between pediatric reproductive tissue and adult donor tissue collection (*p* < 0.05)

All donors in the reproductive fat group were female, again due to the fact that the fat was obtained during spaying (Table 1). Donors of subcutaneous fat obtained from adult cats were approximately 30 % female, leading to a large difference in the sex distribution between the groups that could not be controlled for.

### Harvest Yield

A far greater quantity of tissue was obtained from the subcutaneous procedure with an average of 9 g of tissue obtained from each animal with a large range of values dependent on the surgical procedure at the time of fat procurement and the preference of the individual veterinarian (Table 1). Approximately 20 times less tissue by weight could be obtained from the adipose tissue surrounding the reproductive organs, regardless of the age of the donor. Interestingly there was no difference in the amount of fat retrieved from the reproductive organs of young or old cats. Tissue harvested from the subcutaneous region of each donor was separated into mechanical or enzymatic digestion. Dual digestion protocols could not be tested using the reproductive fat, due to the limited amount of tissue obtained from each donor. Thus, all reproductive fat samples were enzymatically digested.

**Fig. 1:**
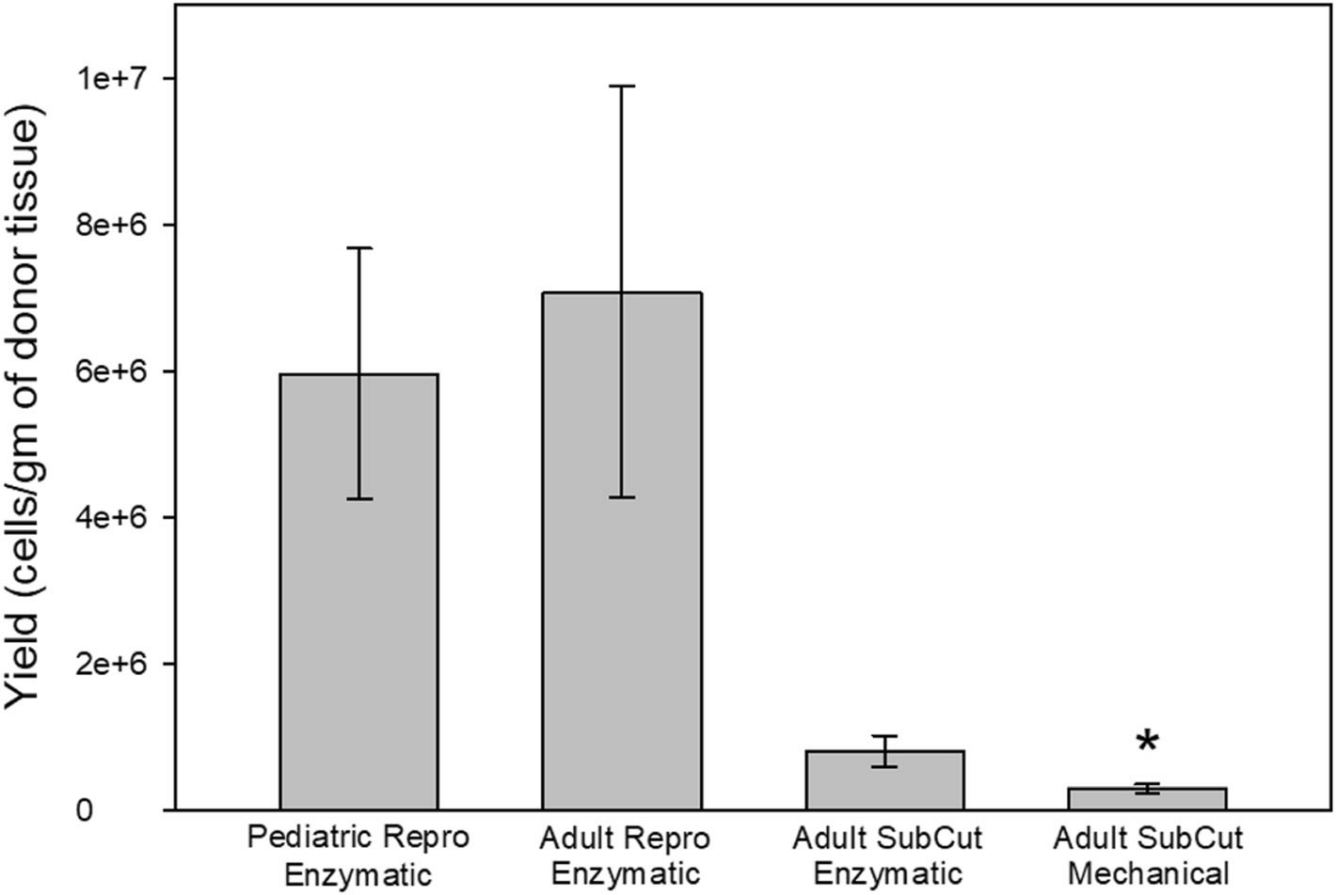
Yield of isolated MSCs from adipose tissue. Following adhesion to plastic, the MCSs were counted and the value divided by the initial weight of the tissue for that sample to obtain the yield. The adipose tissue surrounding the reproductive organs had the highest density of cells per gram of tissue regardless of the age of the donor. * indicates statistical differences (*p* < 0.05) compared to the other groups.

While more total tissue was obtained from the subcutaneous region, the yield of live MSCs per gram of tissue was greater for the reproductive fat. Figure 1 illustrates the vast difference in the yield per gram of tissue in the reproductive fat versus the subcutaneous tissue samples, regardless of the age of the reproductive fat donor. To determine whether a different digestion method could rescue the poor live cell yield in the subcutaneous group, we took samples of subcutaneous fat and mechanically digested it. The method of tissue digestion did not alter the results, with mechanical digestion demonstrating the fewest MSCs per tissue weight compared to the two reproductive groups, but no difference than the enzymatically digested subcutaneous samples. Even with the lower density of cells, the vastly larger amount of starting material retrieved from the subcutaneous fat samples still resulted in a larger total number of cells (Table 1).

### Harvest Viability and Morphology

**Fig. 2:**
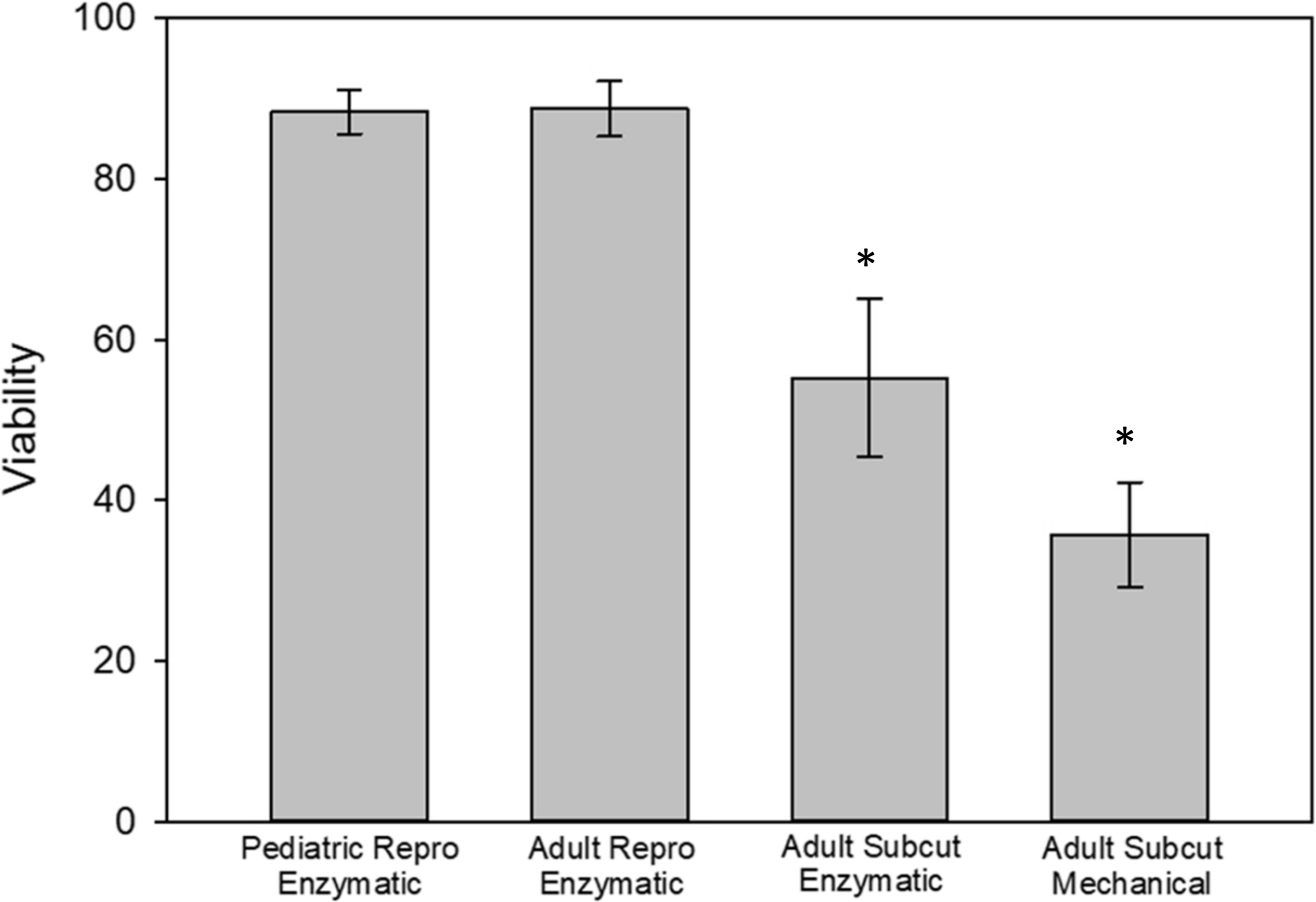
Initial viability of the isolated MSCs. MSCs isolated from reproductive adipose tissue demonstrated significantly higher percentage of viable cells when compared to MSCs isolated from subcutaneous adipose tissue digested either enzymatically or mechanically. * indicates statistical differences (*p* < 0.05) compared to the reproductive enzymatically-digested groups.

Fat cells were plated and 24 h later, the adherent cells were analyzed for viability and morphology. Initial viability following enzymatic digestion of reproductive adipose tissue resulted in over 85 % live cells for both pediatric and adult tissue donors (Fig. 2). In contrast, there was a statistically significant decrease in viability of cells isolated from the subcutaneous tissue. The viability of the cells obtained from subcutaneous fat was approximately 35 % for the mechanical digestion group, while the value was near 55 % for the enzymatic digestion procedure.

**Fig. 3:**
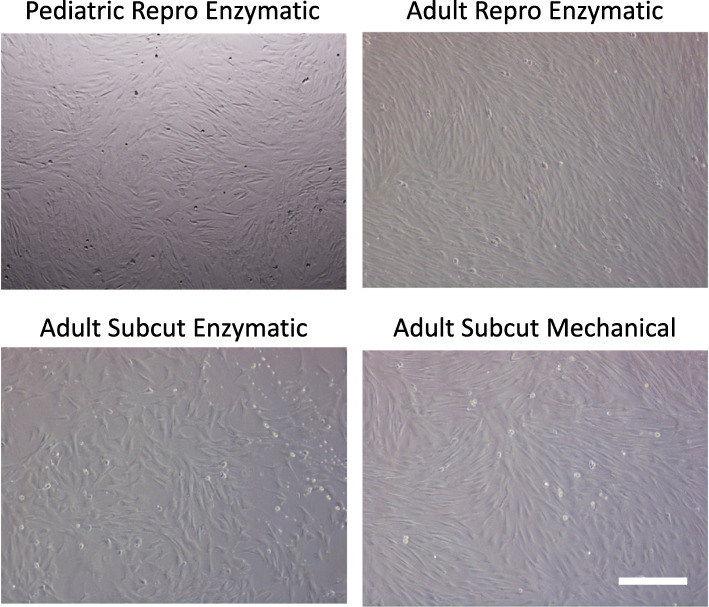
Cell morphology. Following digestion, plated cells that adhered to plastic were cultured to confluency. There were no obvious differences between the groups, although cells from the reproductive group had the most consistent appearance. Scale bar = 200 μm

Cells from the two harvest sites adhered well to plastic and formed highly homogenous monolayers. All cells took on the expected fibroblast-like spindle appearance (Fig. 3) with the cells from reproductive fat showing the most consistency in shape. There were no noticeable morphological differences between the subcutaneous fat that was enzymatically digested versus mechanical digestion.

### Biomarker Expression

**Fig. 4:**
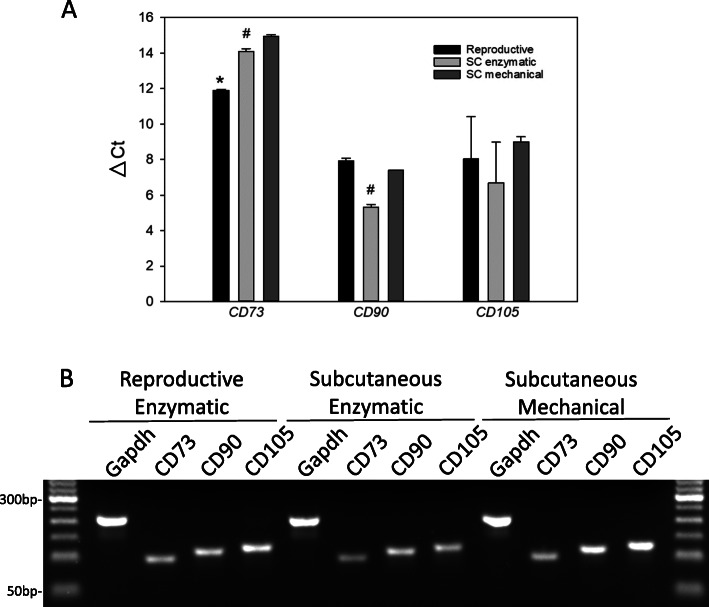
MSC Biomarker Expression. **A**) Standard MSC characterization biomarkers were measured via qRT-PCR. The ΔCT values, normalized to the housekeeping gene *Gapdh*, showed high expression CD73, CD90 and CD105. * indicates a statistically significant difference between the reproductive tissue compared to the other two groups. # indicates statistically significant differences between the subcutaneous enzymatically-digested fat and the other two groups. **B**) Amplicons were run on gels and typical examples shown here with single bands of the anticipated sizes.

Assessment of the extracted RNA demonstrated acceptable quality and concentrations of RNA samples from all groups (Table 2). However, due to limited starting tissue from the reproductive samples, PCR was run on pooled reproductive samples (pediatric and adult). RNA quality met the anticipated 260/280 ratio of > 1.9 for all 3 groups. The 260/230 ratios were less than the anticipated 2.0 but were not statistically different. Verification that the starting material was comprised of MSCs was conducted by examining expression of classic MSC biomarkers using methods previously published [19, 20]. Figure 4 A summarizes the results with high levels of expression of the MSC biomarkers CD73, CD90 and CD105. While CD73 was expressed in the lowest amounts compared to CD90 and CD105, the reproductive fat had the highest expression level of CD73 (lowest ∆Ct value). Subcutaneous enzymatically digested tissue had the highest expression level of CD90. The raw Ct values are provided in supplemental Table 1, confirming the higher expression levels of CD90 and CD105 in all samples. Fragments were run on gels to confirm the correct size (Fig. 4B). The resulting PCR products were purified and sent to Genewiz for sequencing. All results matched the expected sequences and sizes (Table 3).

**Table 2:** RNA Assessment. The quantity and quality of RNA obtained from the 3 sources is provided. Ratios near 2 indicate high quality RNA.

Adipose Source	Digestion Type	RNA Concentration (ng/uL)	260/280 Ratio	260/230 Ratio
Reproductive	Enzymatic	376.2	2.01	1.81
Subcutaneous	Enzymatic	179.6	2.00	1.56
Subcutaneous	Mechanical	352.3	2.00	1.62

**Table 3:** Primer sequences

Primer	Amplicon Size (bp)	Sequence
*Gapdh*	234	Forward: GCGTGAACCACGAGAAGTAT Reverse: CAGTAGAAGCAGGGATGATGTT
*CD73*	112	Forward: CAACATGGGCAACCTGATTTG Reverse: CCGAATGCCACCTCCATTTA
*CD90*	130	Forward: CCTCTCTGCCTGATGAAACTAA Reverse: CAGAGTTCTGGAAGCTCTTAGG
*CD105*	138	Forward: CCTTTGGCGCCTTCCTTAT Reverse: GGTTGGTGCTACTGCTTTCT
*CD34*	199	Forward: ACCATCAAGGGAGAAATCA Reverse: GTCAGTTCCTCCCCATTAC
*Gata4*	251	Forward: CCTCTTGCAATGCGGAAAGA Reverse: GACATCGCACTGACTGAGAAC
*Gata6*	246	Forward: CTCGACCGCTTGCTATGAAA Reverse: GCTCGCTGTTCTCAGGATTAG
*Sox2*	280	Forward: GCAGTACAACTCCATGACCA Reverse: GTAGTGCTGGGACATGTGAA
*Sox17*	322	Forward: CCGCACGGAATTTGAACAGTA Reverse: CCGTTCAAGTGGCAGACAAA
*Pdx1*	343	Forward: GCTACGCAGCTCTACAAGGA Reverse: TAGACTTCATCCACGGGAAAGG

In order to assess differences in the multipotency of the cells, we examined the early differentiation markers GATA4 involved in embryogenesis of the mesoderm and endoderm [21], GATA6 a mesodermal marker involved in regulation of cell differentiation [22], SOX2 essential for pluripotency maintenance in stem cells [23] and SOX17 involved in the regulation of embryonic development [24].

**Fig. 5:**
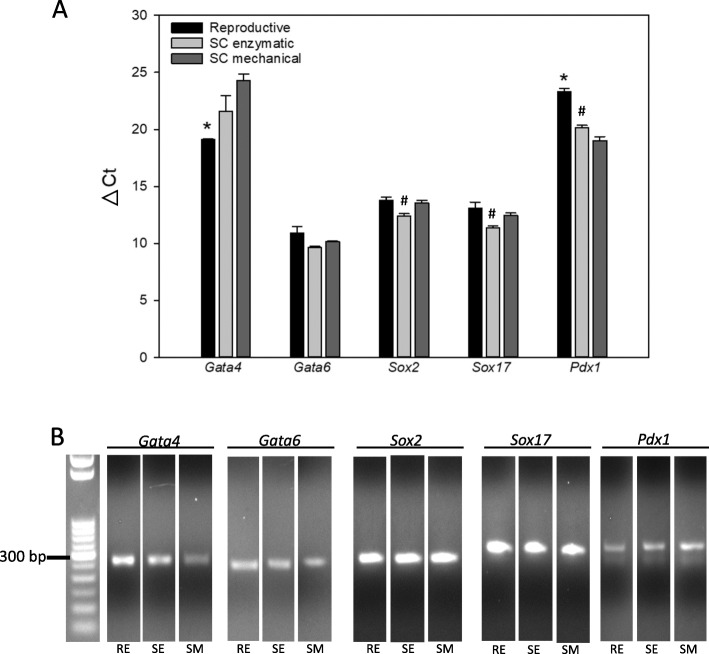
Biomarker Expression. **A**) Five biomarkers for pluripotency, differentiation and expansion were measured via rtPCR. The ΔCT values, normalized to the housekeeping gene *Gapdh*, showed relatively high expression of pluripotent and expansion markers, *Gata6*, *Sox2* and *Sox17*. * indicates a statistically significant difference between the reproductive tissue compared to the other two groups. # indicates statistically significant differences between the subcutaneous enzymatically-digested fat and the other two groups. **B**) Examples of amplicons with single bands of the anticipated sizes are shown. The original gels are provided in Supplemental Figure 1. RE = Reproductive Enzymatic Group, SE = Subcutaneous Enzymatic Group, SM = Subcutaneous Mechanical Group.

*Gata4* expression was low in all 3 samples (Fig. 5 A). with the cells from reproductive fat showing more expression than the other 2 groups (lower ΔCt). As expected, *Gata6* expression was high in the feline cells. The digestion method did affect *Gata6* expression as the enzymatic and the mechanical digestion performed similarly. *Sox2* expression was greatest in the cells digested enzymatically, showing lesser amplification in the mechanically digested samples (Fig. 5 A). Expression of *Sox17* followed the same trend as *Sox2* expression, demonstrating an increase in expression in the enzymatically digested tissue. PDX1 is a marker of early human pancreatic development as is typically only detected in late endoderm differentiation [25]. After failing to detect significant levels of *Gata4* in the feline adipose tissue, we added the analysis of *Pdx1* as a negative control. As expected, *Pdx1* was detected only after extremely high cycle numbers, and normalization to *Gapdh* did not change the results (Fig. 5 A).

Because both Gata4 and Pdx1 were consistently detected but at high cycle levels (> 35 cycles), the fragment sizes were evaluated to ensure that they were the correct size and not primer dimers or contaminants. Figure 5B provides examples of the amplicons showing the anticipated size for all of the biomarkers with no additional bands present. The gels were cropped to provide a standard order for the groups. The full gels are provided in the supplemental section of this manuscript (Figure S1). In addition, the amplicons were sequenced showing a match with the expected results (Table 3). In order to appreciate the level of expression, the raw Ct values are provided in Supplemental Table 2.

### Cell Culture Expansion

**Fig. 6:**
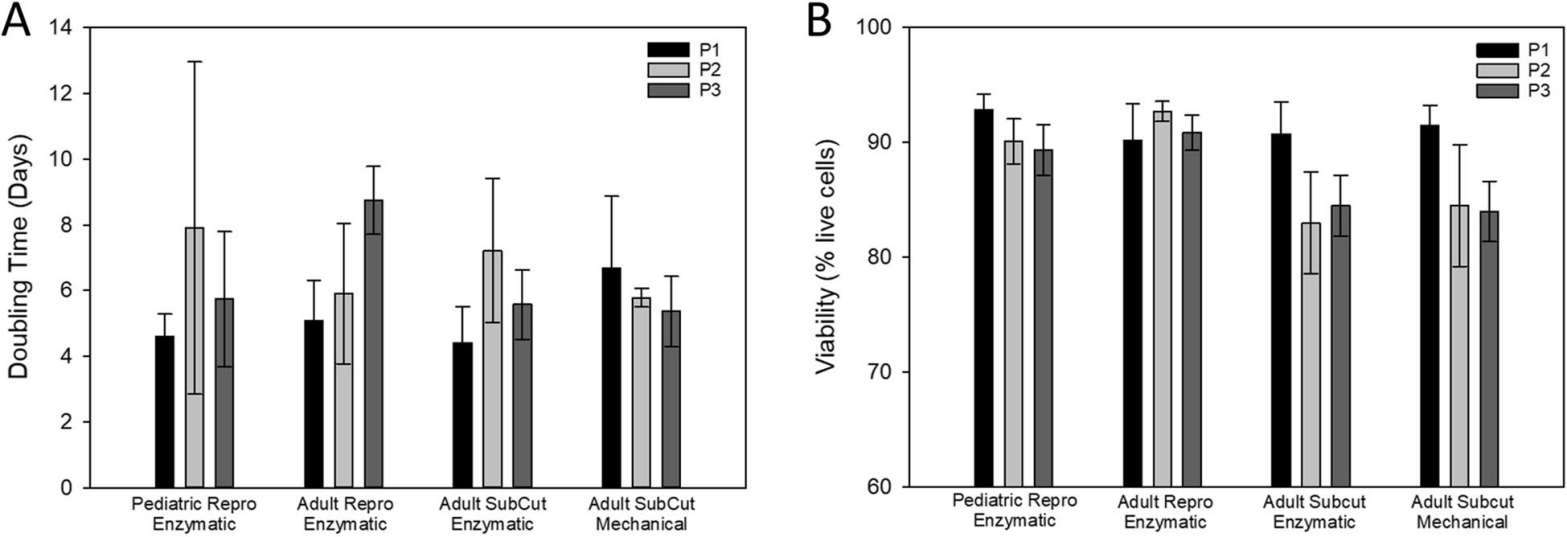
Cell Expansion. **A**) The doubling time for the 3 groups at P1 shows high variability and no statistical differences between groups. P3 doubling time showed less variability with no statistical differences between or within groups. **B**) Viability was high for all groups for the P1 passage. With more passages, the reproductive fat groups had the most consistently high viability.

To measure any effects of the different donor characteristics on cell culture expansion, cells were seeded at an approximate density of 5,000 cells/cm^2^ and cell expansion measured by population doubling times. Figure 6 A summarizes the differences in expansion capability for the four groups. During P1-P3, there were no significant differences between the groups’ mean doubling time. Interestingly, all groups showed a trend towards longer doubling times with subsequent passages, except for the cells obtained from adult cat subcutaneous tissue and mechanically digested, which had a slight decrease in doubling time with each passage.

Viability of the cells was measured at the end of each passage. The cells in these groups showed high viability with no differences between groups (Fig. 6B).

## Discussion

MSCs are becoming increasingly relevant to clinical care [5] but the outcomes associated with MSC treatment have shown great inconsistency. Some of the outcome variability may be due to the altered characteristics associated with the donors such as age and sex and based on the site of isolation of the initial tissue [26]. Other factors responsible for inducing variability may include the methods of isolation, the amount of tissue harvested, and the passage number. In felines, there have been few studies to compare sources of MSCs and they have focused on comparing bone marrow-derived MSC to adipose MSCs [18]. Further, when working with abdominal adipose tissue, the exact donor site often is not clarified in the reports [27, 28]. A unique study design was undertaken to enhance the translation of the data to the clinical setting. The tissue was retrieved during veterinary standard procedures, either abdominal surgery or spaying of female cats and, when possible, the settings were either licensed clinical veterinary practices or animal shelters.

Obviously, a much greater quantity of adipose tissue can be harvested from the subcutaneous fat depot as compared to the reproductive depot. However, the density of MSCs obtained from those regions was vastly different with the tissue surrounding the reproductive organs resulting in 40 times more MSCs per tissue weight. Differences in the age of the female cats had no effect on the yield of cells from reproductive fat. Even with this great difference in yield (cells per tissue weight), the overall number of cells obtained was greater with the subcutaneous harvest due to the large volume of subcutaneous fat that could be retrieved. However, subcutaneous cells had an extremely low viability, especially when digested mechanically, along with the slowest P2 doubling time. The range of doubling times reported here are consistent with some previous publications of feline abdominal adipose tissue [29] but slower than those reported by others [30, 31].

Only one other group has focused on different fat depots in felines comparing abdominal from subcutaneous locations [27]. They found that there was no difference between the two locations in the multipotent markers nor in the ability of the cells to differentiate into mesoderm lineages. However, they did not identify the exact location of the abdominal fat depot, thus it is difficult to directly compare their results to those reported here.

There have been reports suggesting that mechanical digestion can be advantageous to enzymatic digestion when small quantities of MSCs are desired due to its decreased cost and time commitment but with a decreased yield [32]. We confirmed that the yield for the mechanically digested sample was significantly less than the other 2 groups and the resulting viability of the cells was poor (approximately 25 %). While the remaining cells could be rescued and passaged, the poor initial outcome indicates that a significantly larger amount of starting material would be necessary to harvest, if using mechanical digestion.

The quality and amount of RNA extracted from the groups showed no relevant differences. This is important because previous studies concluded that fat contains a high fatty acid content that can interfere with the extraction of pure RNA, indicated by the 260/230 ratio [33]. We were able to confirm the identity of the starting cells as MSC based on their biomarker signature described by previous publications [18, 19, 27, 29, 31]. While all three groups presented with high levels of expression of standard MSC biomarkers, the reproductive fat sample had the highest level of CD73 while the subcutaneous enzymatic sample had more CD90, although these differences were likely not clinically relevant. We further investigated the pluripotency and lineage assignment potential to further characterize the tissue using previously published techniques [19, 27]. While the differences in expression levels of some of the pluripotent biomarkers reached statistical significance, the differences were small and likely not biologically relevant. Importantly, all of the pluripotency biomarkers were detected at high CT values, indicating low levels of gene expression.

SOX2 is a master stem cell transcription factor, determining the stemness capacity of cells [23]. While high levels of expression of *SOX2* have been reported in human, monkey and canine abdominal fat previously [34, 35], its detection in feline cells has been variable. Cat embryos, along with undifferentiated feline neural stem cells, contain high levels of *Sox2* [36–38]. In addition, a number of labs have shown that induced pluripotent stem cells derived from feline fibroblasts express *Sox2* [39, 40]. In comparison with canine cells, feline amniotic MSCs showed a much lower expression of *Sox2* and less immunostaining of the protein compared to canine amniotic stem cells [19]. Some have reported the inability to detect *Sox2* or the resulting protein in feline abdominal or subcutaneous adipose MSCs [27, 28]. In contrast, our results are consistent with those of Lee et al., showing detection of *Sox2* in the abdominal adipose tissue [31]. Unfortunately, ours is the only study to include the raw Ct values of our results rather than relative values that provide no indication of the absolute levels.

*GATA6* encodes a transcription factor normally found in MSCs essential to the self-renewal properties of pluripotent cells. Knockdown of *GATA6* results in suppression of the self-renewal capacity of MSCs [41]. In cats, *Gata6* is assumed to be involved in differentiation similar to humans, but that has yet to be examined. Studies have described the presence of *Gata6* in feline induced pluripotent stem cells [42] and in cat embryos [36], but ours is the first study to report the presence of *Gata6* in feline adipose MSCs.

SOX17, identified in human adipose derived MSCs [43], is involved in the capacity of the cells to differentiate into endoderm and eventually to hepatocytes [44] or pancreatic tissue [33]. Like *Gata6*, *Sox17* has been detected in feline induced pluripotent stem cells [42], but has not been investigated in feline adipose MSCs. Thus, this is the first study to identify *Sox17* in feline adipose-derived MSCs.

*GATA4* is found in high levels in human ovaries where it is associated with sex determination along with ovary growth and function, but not in adipocytes [45]. GATA4 is involved in differentiation into osteoblasts and development of the heart and has been reported to be expressed in only 15 % of rat bone marrow-derived MSCs based on immunohistochemistry [21]. Thus, it was not surprising that little was detected in the adipose MSCs. In fact, minimal expression confirmed that ovarian tissue did not contaminate the reproductive fat group. Likewise, PDX1 is a biomarker of pancreatic development and is farther down the differentiation lineage. We included *Pdx1* detection as a negative control, thus the lack of *Pdx1* was expected.

A major limitation of the study was the fact that because the reproductive fat was obtained during spaying, there was a difference in the sex distribution between groups. Based on a clinically relevant study design that collected excess tissue from standard procedures on predominantly community dwelling cats, sex could not be controlled for as a variable. A much larger clinical study is warranted to determine possible sex-based differences. In addition, this study did not analyze the purity or differentiation capacity of the cells at any of the passages.

While cells from all three groups studied here had similar pluripotency profiles, the improved yield (cells/tissue weight) and initial cell viability was dramatically better for the cells harvested from the reproductive tissue, which can be harvested ethically during spaying procedures. If the goal is an autologous product, then reproductive fat harvests would not be appropriate, but the field is quickly moving toward the allogeneic model. Consequently, fat surrounding the reproductive organs may offer a consistently high-quality source of MSCs. In addition, we showed that enzymatic digestion was the best approach for subcutaneous tissue and the only option available when working with reproductive tissue due to the small amount of starting material.

## Conclusions

In conclusion, the work summarized here demonstrates that feline reproductive adipose tissue is a reasonable source of MSCs to be cultured for eventual therapeutic application compared to a subcutaneous fat depot. *The age of the donor did not affect the quality of the MSCs from the reproductive fat.* The MSCs should be isolated via predominantly enzymatic digestion, but further research should be done to continue optimizing a digestion protocol. For example, in certain clinical situations, long-term cell culture may not be possible and mechanically-digested subcutaneous fat may be the only option. In addition, further research should continue to further characterize feline adipose-derived MSC, including the differentiation potentials.

## Methods

### Adipose Tissue Collection

The adipose tissue was collected from 34 healthy female cats, including cats undergoing spaying at local animal shelters and the discarded tissue removed during spaying (the ovaries and uterine horn) was collected and the fat dissected. Subcutaneous fat was removed from male and female cats under anesthesia for unrelated surgical procedures. Additionally, two samples were obtained from research animals undergoing euthanasia (sodium pentobarbital) at the completion of an unrelated study. The University of Kansas Medical Center’s IACUC determined that tissue removed during spaying or after euthanasia was exempt from required protocol approval. As all tissue collected would otherwise have been discarded, no consent was necessary. The licensed veterinarians overseeing the animals approved the collection of the discarded tissue. No exclusion criteria were set for the study. Table 1 summarizes the donor characteristics. All donors were standard long or shorthair cats and were fully immunized at the time of tissue retrieval. Due to the fact that tissue was retrieved during other procedures, the site of tissue collection (reproductive versus subcutaneous) could not be randomized. Group assignments were based on convenience of the surgical procedures completed through the duration of the study. Due to large differences in the tissue mass obtained from the different sites, blinding of the initial sample groups could not be done. Analysis of the later passages was completed in a blinded fashion. Donor characteristics were provided by the attending veterinarian and included age, weight, sex, body condition, vaccination history, and general health status including feline leukemia virus results.

For subcutaneous fat collection, donors were positioned in dorsal recumbency and the surgical site was prepared using standard aseptic surgical procedures. A 1 cm length incision was made at the midline to allow access to fat deposits in the ventral abdomen. Using a Tulip multi-port tissue harvester cannula attached to a 60CC syringe the abdominal fat was broken up with gentle back and forth movement of the cannula device. The syringe was then used to draw out the fat tissue that was transferred to collection media, consisting of Dulbecco’s Modified Eagle’s Medium (DMEM) supplemented with 1 % pen strep.

Reproductive adipose tissue was collected aseptically during standard spaying of healthy cats. The removed ovaries and uterine horn were placed in chilled transport medium comprised of DMEM/F12 (ThermoFisher, Cat#124,000,024) containing 1 % Penicillin-Streptomycin (ThermoFisher, Cat#15,140,122) and transported to the lab on ice. Under aseptic conditions, the fat was manually dissected from the reproductive organs.

### Digestion of Adipose Tissue

### Mechanical Digestion

Under aseptic conditions, the tissue was rinsed, weighed, and minced using sterile scissors. Mechanical digestion involved passing the adipose tissue between two 60 mL syringes connected with an anaerobic luer-to-luer connector (Tulip, Cat #TP ATLLLL2.4). Using the 2.4 mm Tulip connector, the tissue was passed between the two syringes 10 times and then passed through the smaller 1.2 mm Tulip connector (Tulip, Cat#TP ATLLLL1.2) another 10 times. The tissue solution was centrifuged at 550xg for 10 min. The cell pellet was reserved for plating in DMEM culture medium. The remaining supernatant was collected and the digestion was repeated twice more with increasing centrifugation speeds in each round of digestion (620xg and 690xg, respectively). The cells from the pellets from each digestion were collected and counted. The pellets were resuspended in DMEM:F12 with 10 % FBS (ThermoFisher, Cat #26,140,087) and 1 % Penicillin-Streptomycin and cultured at 37 °C, 5 % CO_2_ in a humidified chamber.

### Enzymatic Digestion

Under aseptic conditions, the tissue was rinsed, weighed, and minced using sterile scissors and digested in a 0.6 WU/mL Liberase (Roch, Liberase MNP-S, /cat #06297790001) for 30 min at 37 °C. For the larger tissue amounts of subcutaneous fat, the tissue was passed through a 100 mm filter and centrifuges at 260xg for 5 min. The supernatant was removed and mixed with 5 mL of red blood cell lysis buffer (Millipore Sigma, Cat #11,814,389,001) followed by centrifugation at 260xg for 5 min. The final supernatant was resuspended in DMEM:F12 with 10 % FBS and 1 % Penicillin-Streptomycin and cultured at 37 °C, 5 % CO_2_ in a humidified chamber.

### Cell Culture

Following digestion, cells were incubated for 48 h at 37 °C and 5 % CO_2_ after which the non-adherent cells were removed, and fresh medium was added. The remaining plastic-adherent cells were trypsinized (Worthington Cat# 9002-07-7) and re-plated at a density of 5,000 cells/cm^2^ into T175 flasks. Cell numbers were calculated using automated cell counter (EVE; NanoEnTek) at the time of passage and during expansion in order to calculate the doubling time. Cells were passaged when they met the requirement of 80–100 % confluency. Flasks that failed to obtain 80 % confluency were removed from analysis. Images of cultured cells were obtained using an Axio Vert.A1 Inverted microscope (Zeiss) with a 10X objective. Cells were not cultured beyond 5 passages.

Doubling time was calculated based on a published protocol [46].

$$\mathrm{DT}\:=\:\mathrm T\ast\ln2/\ln(\mathrm{Xe}/\mathrm{Xb}-1)$$

T is time, ln is the natural log, Xb indicates the starting cell number, and Xe indicates the ending cell number.

### Viability

Viability was measured from fluorescence emission levels of calcein (ThermoFisher, Cat #C3099) and propidium iodide (ThermoFisher, Cat #P3566) on a multi-mode plate reader (BioTek Cytation 5). Calcein stains live cell green and PI stains dead cells as red. Viability was calculated as:

$$\lbrack\mathrm{live}\;\mathrm{cell}\;\mathrm{emission}\;(\mathrm{green})/\mathrm{total}\;\mathrm{cells}\;(\mathrm{green}\:+\:\mathrm{red})\rbrack\;\mathrm x\;100$$

Trypan blue exclusion staining was also used as a measure of viability. The cells were dispersed into single cell suspensions using trypsin and briefly incubated with Trypan blue. Viability was determined as the ratio of (live cells / total number of cells) x 100.

### Biomarker Expression

Total RNA was extracted from frozen cell pellets of the digested adipose tissue using the mirVana RNA Extraction kit (ThermoFisher, Cat#AM1560) per the manufacturer’s protocol. RNA quality was assessed using the NanoDrop 1000 Spectrophotometer (Thermo-Fisher). Absorbance was read at 230, 260 and 280 nm. The 260/280 ratio of ~ 2.0 indicates high quality RNA. Likewise, a 260/230 ratio of between 2.0 and 2.2 indicates a pure sample.

One µg of total RNA was used to make cDNA using SuperScript IV Reverse Transcriptase (Invitrogen, Cat #18,090,010) as per the manufacturer’s protocol using oligo (dT). Primer sequences for the biomarkers were determined using BLAST (Table 3) and confirmed via gel electrophoresis.

The cDNA was run through RT-qPCR on the Applied Biosystems ViiA7 PCR System using Power SYBR Green PCR Master Mix (Applied Biosystems, Cat #4,367,659) using the following protocol: 2 min at 95℃ for initial denaturation, followed by cycling 15 s at 95℃ for denaturation, 10 s at 60℃ for annealing, and 60 s at 70℃ for extension. This protocol repeated for 49 cycles, followed by recording a melt curve. All samples were amplified in triplicate. An automatic threshold was utilized and a cut off of 40 cycles was determined to indicate presence or absence of biomarkers. Negative controls without template were run to 50 cycles with no amplification. The cycle threshold values were normalized to Gapdh. To confirm the results, amplicons were run on gels to confirm the correct sizes. In addition, the resulting PCR products were purified and sent to Genewiz for Sanger sequencing. All results matched the expected sequences and sizes (Table 3).

### Statistical Analysis

Data were analyzed using a one-way analysis of variance (ANOVA) on ranks with post hoc Dunn’s pairwise comparison was performed using SigmaPlot 13.0. *P* values of less than 0.05 were considered statistically significant, noted by symbols in the graphs. Data are presented as averages ± SEM.

## Supplementary Information

**Additional file 1: Figure S1**The gels shown in Figure 5B were cropped from the original gels shown here, which included amplicons for this study as well as another study comparing different types of serum, indicated as “Other Study” in the figure.

**Additional file 2:**

**Additional file 3:**

## Data Availability

Data generated and analyzed for the current study are available from the corresponding author.

## Abbreviations

CO_2_: Carbon dioxide.
cm^2^: centimeter squared
CD: Cluster of differentiation
Ct: threshold cycle
ΔCt: Change in Ct
dT: deoxythymine
DT: doubling time
DMEM/F12: Dulbecco’s Modified Eagle Medium F-12
g: grams
mm: millimeter
MSC: multipotent stromal cells
P1, P2 or P3: passage number
RE: reproductive enzymatic
RT-qPCR: Quantitative reverse transcription polymerase chain reaction
RNA: ribonucleic acid
SM: subcutaneous mechanical
SE: subcutaneous enzymatic
T: Time
WU: Wunsch units
Xb: Starting cell number
Xe: Ending cell number
Xg: times gravity
