# Supplementary figures and images for: Viability, yield and expansion capability of feline MSCs obtained from subcutaneous and reproductive organ adipose depots
Source: BMC Vet Res. 2021 Jul 15;17:244. doi: 10.1186/s12917-021-02948-0 (PMC8281647; doi:10.1186/s12917-021-02948-0)

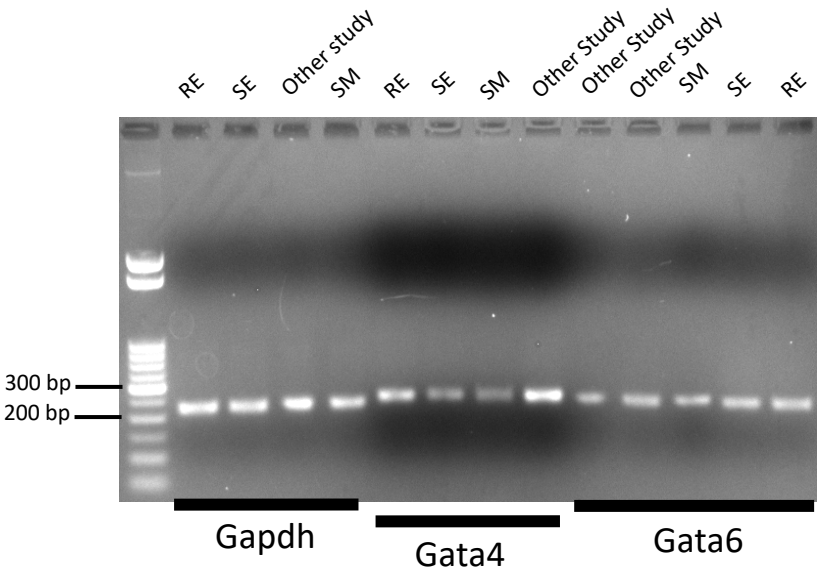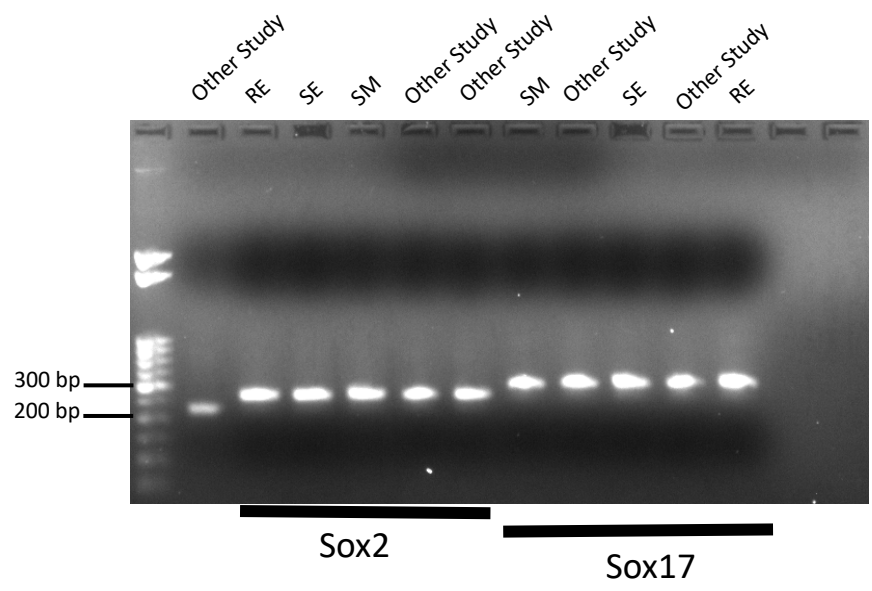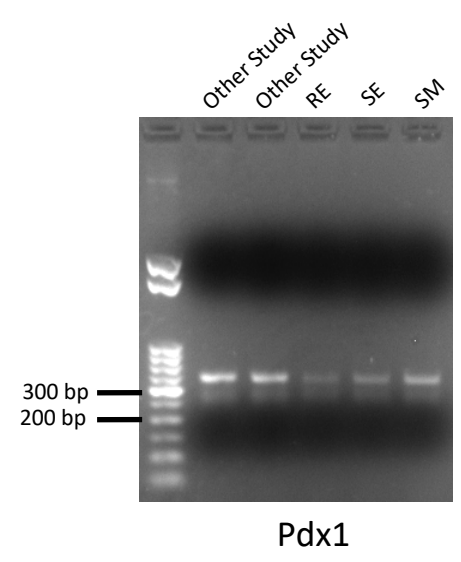

Supplement: Supplementary file 1 — Additional file 1: Figure S1The gels shown in Figure 5B were cropped from the original gels shown here, which included amplicons for this study as well as another study comparing different types of serum, indicated as “Other Study” in the figure. [file 12917_2021_2948_MOESM1_ESM.pdf]
