# Supplementary material for: Viability, yield and expansion capability of feline MSCs obtained from subcutaneous and reproductive organ adipose depots
Source: BMC Vet Res. 2021 Jul 15;17:244. doi: 10.1186/s12917-021-02948-0 (PMC8281647; doi:10.1186/s12917-021-02948-0)
Supplement: Supplementary file 2 — Additional file 2: [file 12917_2021_2948_MOESM2_ESM.docx]

**Supplemental Table 1. MSC Identity**

Raw Ct values indicate the detection cycle of the following genes from 3 independent trials run in triplicate.

| **Adipose Source** | **Digestion** | ***Gapdh*** | ***CD73*** | ***CD90*** | ***CD105*** |
| --- | --- | --- | --- | --- | --- |
| Reproductive | Enzymatic | 18.55±0.08 | 30.45±0.11 | 26.45±0.15 | 26.58±2.31 |
| Subcutaneous | Enzymatic | 22.12±0.10 | 36.20±0.10 | 27.44±0.07 | 28.82±2.30 |
| Subcutaneous | Mechanical | 17.86±0.05 | 32.79±0.14 | 25.25±0.11 | 26.28±0.29 |
