# Supplementary material for: Viability, yield and expansion capability of feline MSCs obtained from subcutaneous and reproductive organ adipose depots
Source: BMC Vet Res. 2021 Jul 15;17:244. doi: 10.1186/s12917-021-02948-0 (PMC8281647; doi:10.1186/s12917-021-02948-0)
Supplement: Supplementary file 3 — Additional file 3: [file 12917_2021_2948_MOESM3_ESM.docx]

**Supplemental Table 2. Multipotent Biomarker Detection**

Raw Ct values indicate the detection cycle of the following genes from 3 independent trials run in triplicate.

| **Adipose Source** | **Digestion** | ***Gapdh*** | ***Gata4*** | ***Gata6*** | ***Sox2*** | ***Sox17*** | ***Pdx1*** |
| --- | --- | --- | --- | --- | --- | --- | --- |
| Reproductive | Enzymatic | 20.76±0.21 | 39.86±0.24 | 31.64±0.08 | 34.57±0.06 | 33.86±0.37 | 44.63±0.27 |
| Subcutaneous | Enzymatic | 22.54±0.12 | 42.83±1.11 | 32.17±0.51 | 34.95±0.56 | 33.89±0.51 | 43.76±0.40 |
| Subcutaneous | Mechanical | 22.63±0.09 | 46.71±1.92 | 32.78±0.66 | 36.19±0.99 | 35.07±0.96 | 43.90±1.39 |
